# Multi-Level Oncological Management of a Rare, Combined Mediastinal Tumor: A Case Report

**DOI:** 10.3390/curroncol32080423

**Published:** 2025-07-28

**Authors:** Vasileios Theocharidis, Thomas Rallis, Apostolos Gogakos, Dimitrios Paliouras, Achilleas Lazopoulos, Meropi Koutourini, Myrto Tzinevi, Aikaterini Vildiridi, Prokopios Dimopoulos, Dimitrios Kasarakis, Panagiotis Kousidis, Anastasia Nikolaidou, Paraskevas Vrochidis, Maria Mironidou-Tzouveleki, Nikolaos Barbetakis

**Affiliations:** 1 Thoracic Surgery Department, “Theageneio” Cancer Hospital, 54639 Thessaloniki, Greece; theocharidisvassilis@gmail.com (V.T.); drgogakos@hotmail.com (A.G.); demtros@yahoo.gr (D.P.); axlazopoulos@hotmail.com (A.L.); mkoutourini@hotmail.com (M.K.); myrtwtzinevi@gmail.com (M.T.); nibarbet@yahoo.gr (N.B.); 2 Second Department of Medical Oncology, “Theageneio” Cancer Hospital, 54639 Thessaloniki, Greece; k.vildiridi@gmail.com (A.V.); ksdim@yahoo.com (P.D.); dimkasar@yahoo.gr (D.K.); 3 Pathology Department, “Theageneio” Cancer Hospital, 54639 Thessaloniki, Greece; pkousidis@protonmail.com (P.K.); nikolaidou.anastasia@yahoo.com (A.N.); 4 ENT Department, General Hospital of Serres, 62100 Serres, Greece; paref25@gmail.com; 5 First Laboratory of Pharmacology, Medical School, “Aristotle” University of Thessaloniki, 54124 Thessaloniki, Greece; mmyronidauth@gmail.com

**Keywords:** mediastinal, tumor, thoracic, oncology

## Abstract

Malignant mediastinal tumors are a group representing some of the most demanding oncological challenges for early, multi-level, and successful management. The timely identification of any suspicious clinical symptomatology is urgent in achieving an accurate, staged histological diagnosis, in order to follow up with an equally detailed medical therapeutic plan (interventional or not) and determine the principal goals regarding efficient overall treatment in these patients. We report a case of a 24-year-old male patient with an incident-free prior medical history. An initial chest X-ray was performed after the patient reported short-term, consistent moderate chest pain symptomatology, early work fatigue, and shortness of breath. The following imaging procedures (chest CT, PET-CT) indicated the presence of an anterior mediastinal mass (meas. ~11 cm × 10 cm × 13 cm, SUV: 8.7), applying additional pressure upon both right heart chambers. The Alpha-Fetoprotein (aFP) blood levels had exceeded at least 50 times their normal range. Two consecutive diagnostic attempts with non-specific histological results, a negative-for-malignancy fine-needle aspiration biopsy (FNA-biopsy), and an additional tumor biopsy, performed via mini anterior (R) thoracotomy with “suspicious” cellular gatherings, were performed elsewhere. After admission to our department, an (R) Video-Assisted Thoracic Surgery (VATS) was performed, along with multiple tumor biopsies and moderate pleural effusion drainage. The tumor’s measurements had increased to DMax: 16 cm × 9 cm × 13 cm, with a severe degree of atelectasis of the Right Lower Lobe parenchyma (RLL) and a pressure-displacement effect upon the Superior Vena Cava (SVC) and the (R) heart sinus, based on data from the preoperative chest MRA. The histological report indicated elements of a combined, non-seminomatous germ-cell mediastinal tumor, posthuberal-type teratoma, and embryonal carcinoma. The imminent chemotherapeutic plan included a “BEP” (Bleomycin^®^/Cisplatin^®^/Etoposide^®^) scheme, which needed to be modified to a “VIP” (Cisplatin^®^/Etoposide^®^/Ifosfamide^®^) scheme, due to an acute pulmonary embolism incident. While the aFP blood levels declined, even reaching normal measurements, the tumor’s size continued to increase significantly (DMax: 28 cm × 25 cm × 13 cm), with severe localized pressure effects, rapid weight loss, and a progressively worsening clinical status. Thus, an emergency surgical intervention took place via median sternotomy, extended with a complementary “T-Shaped” mini anterior (R) thoracotomy. A large, approx. 4 Kg mediastinal tumor was extracted, with additional RML and RUL “en-bloc” segmentectomy and partial mediastinal pleura decortication. The following histological results, apart from verifying the already-known posthuberal-type teratoma, indicated additional scattered small lesions of combined high-grade rabdomyosarcoma, chondrosarcoma, and osteosarcoma, as well as numerous high-grade glioblastoma cellular gatherings. No visible findings of the previously discovered non-seminomatous germ-cell and embryonal carcinoma elements were found. The patient’s postoperative status progressively improved, allowing therapeutic management to continue with six “TIP” (Cisplatin^®^/Paclitaxel^®^/Ifosfamide^®^) sessions, currently under his regular “follow-up” from the oncological team. This report underlines the importance of early, accurate histological identification, combined with any necessary surgical intervention, diagnostic or therapeutic, as well as the appliance of any subsequent multimodality management plan. The diversity of mediastinal tumors, especially for young patients, leaves no place for complacency. Such rare examples may manifest, with equivalent, unpredictable evolution, obliging clinical physicians to stay constantly alert and not take anything for granted.

## Simple Summary

Modern clinical oncology faces several challenges in the diagnosis and management of large mediastinal malignant tumors, both mixed and non-mixed. Early detection is crucial to improve survival rates, as these tumors often present with non-specific symptoms or are detected incidentally. Successful surgical intervention plays a key role in treatment, yet it remains complicated by the tumor’s location and the involvement of adjacent structures. Postoperative oncological management, including radiation and chemotherapy, is vital to prevent recurrence. A multidisciplinary approach involving thoracic surgeons, oncologists, radiologists, and pathologists is essential for optimal outcomes, ensuring precise diagnosis, tailored treatment plans, and effective follow-up. Cooperation between these specialties enhances patient prognosis and quality of life, making integrated care the cornerstone of modern oncology in mediastinal malignancies.

## 1. Introduction

Malignant mediastinal tumors remain a group of the most demanding oncological challenges for early, multi-level, and successful treatment. Arising from a variety of tissues within the mediastinum, these tumors encompass a heterogeneous spectrum, including both benign and malignant entities. Their low incidence contributes to limited clinical familiarity, often leading to delays or difficulties in diagnosis. Apart from the necessity for precise and thorough histological identification, as soon as possible, the possibility of eventually detecting combined cancerous entities increases the level of difficulty regarding direct and successful therapeutic treatment [1]. Combined tumors, consisting of two or more distinct histologic components, are exceptionally uncommon and introduce further complexity, while the biological behavior of each component must be considered individually. The overall clinical management is rather complex due to their peculiar topographic anatomical location and frequent association with aggressive histological features [2]. Furthermore, complications, such as vascular compression, pleural effusions, or immune suppression, secondary to chemotherapy, are usually accompanied with poor prognosis and life expectancy [3]. This essay presents a detailed overview of the current diagnostic pathways and therapeutic strategies used against combined mediastinal tumors, based on a rare clinical case of a 24-year-old male. Emphasis is placed on interdisciplinary coordination and collaboration and the initial and intermediate surgical interventions, as well as the complications that emerged. Such cases frequently require a nuanced diagnostic approach, integrating advanced imaging, histopathologic assessment, and, at times, molecular analysis. From a clinical perspective, the rarity and heterogeneity of these tumors complicate the establishment of standardized management protocols, underscoring the need for individualized treatment strategies and multidisciplinary care. This case reinforces the need for extensive tissue sampling and, when available, adjunct molecular profiling to guide both classification and management strategies, as well as the necessity for vigilant follow-up.

## 2. Clinical Presentation

We report a case of a 24-year-old male with an incident-free prior medical general and oncologic history. The patient began to suffer from early work fatigue, progressive chest discomfort and pain, dyspnea on exertion, orthopnea, and intermittent fever episodes. Physical examination revealed dullness to percussion over the (R) lower lung field, decreased breath sounds, bronchial (R) hemithorax breathing, and tachycardia. This pathological symptomatology led to an initial chest X-ray, which indicated the existence of a large mediastinal mass [4].

**Figure 1:**
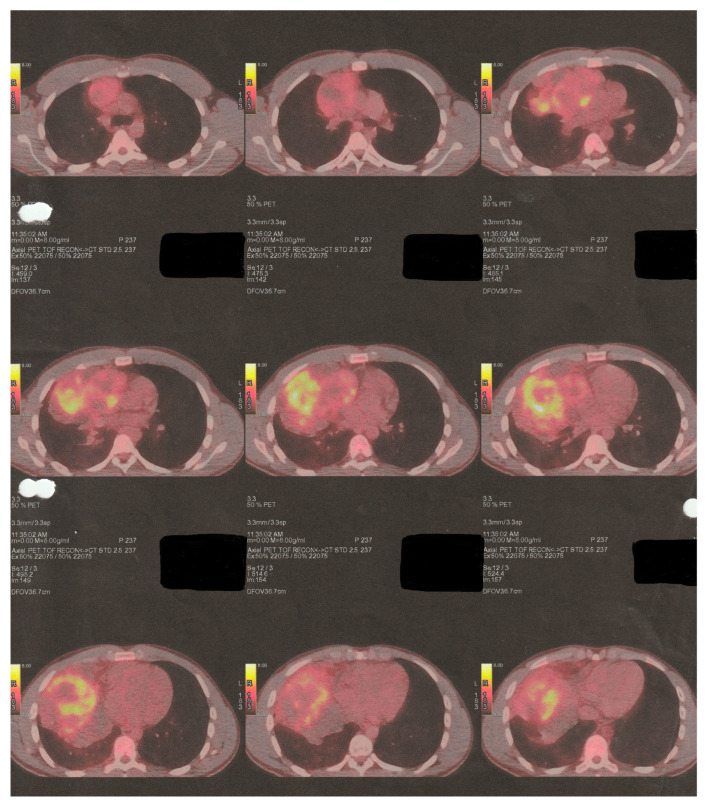
Initial preoperative PET-CT imaging control.

The following imaging procedures that took place, such as high-resolution chest computer tomography (chest CT) and positron emission tomography–computed tomography (PET-CT), confirmed the presence of an heterogenous anterior and middle mediastinal voluminous formation, extending into the (R) lung cavity, with quite notable size measurements (DMax: ~11 cm × 10 cm × 13 cm), a significant standardized uptake value (SUV: 8.7), areas of cystic degeneration, necrosis, and associated pleural effusion (Figure 1).

The tumor applied additional pressure on both (R) heart chambers, causing tracheobronchial compression and mediastinal shift. The PET-CT imaging process identified hypermetabolic foci in mediastinal lymph nodes and neighboring osseous structures, suggesting additional metastatic spread.

**Figure 2:**
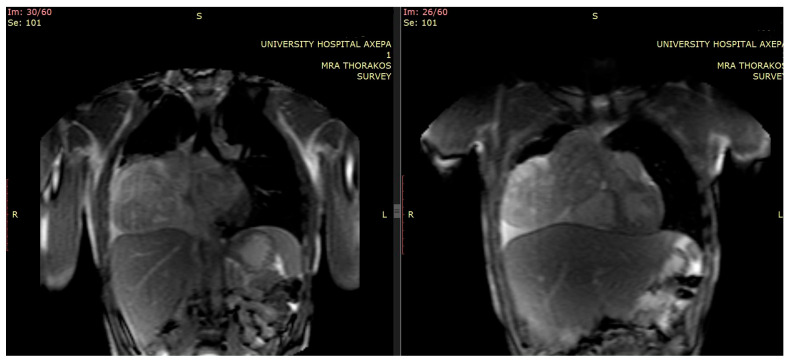
Initial preoperative MRI.

Furthermore, additional magnetic resonance imaging (MRI) provided details regarding local invasion, particularly about pericardial and vascular structures [5] (Figure 2).

After detailed laboratory control, regarding the investigation of tumor markers, aFP levels were calculated to be over 50 times greater than the normal range, reaching values over 2600 ng/mL, while beta-human Chorionic Gonadotropin (β-HCG: 42 IU/L) and Lactate Dehydrogenase (LDH: 839 U/L) were modestly raised. Other findings demonstrated leukocytosis, anemia, thrombocytosis, and mild transaminasemia. Serum tumor marker trends over time, including aFP, β-HCG, LDH, CEA, and CA 19-9, with reference ranges provided for clinical context, are presented.

The need for immediate histological–pathological confirmation led to the decision to perform two consecutive diagnostic attempts, resulting in non-specific histological findings [6]. The first attempt was conducted via fine-needle aspiration (FNA) biopsy, with the results negative for malignancy indications. Subsequently, an additional tumor biopsy, performed via mini anterior (R) thoracotomy, detected restricted “suspicious” cellular gatherings. The abovementioned procedures took place elsewhere [7].

Immediately after the repetitive results, the patient was admitted to our department. A (R) Video-Assisted Thoracic Surgery (VATS) procedure was performed [8], with direct multiple sampling of tumor tissue, while moderate accompanying pleural effusion was drained [9].

**Figure 3:**
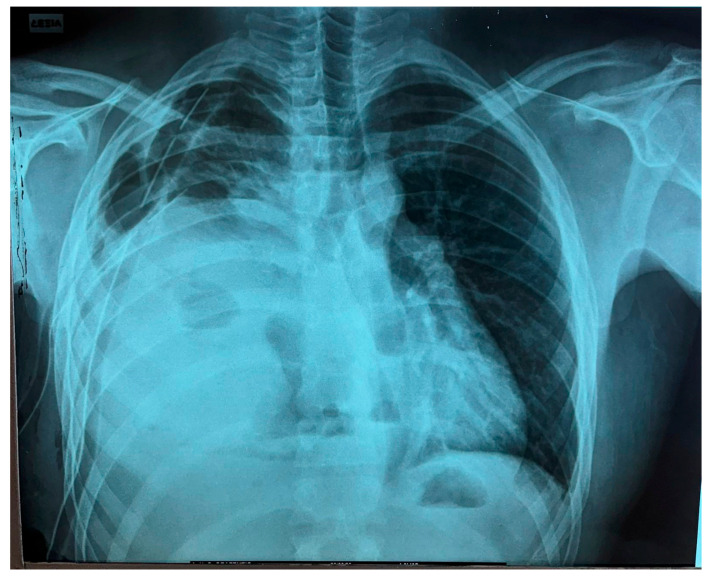
First postoperative day X-ray control (after VATS R).

The tumor’s measurements had increased (DMax: 16 cm × 9 cm × 13 cm). Intraoperatively, severe degree of atelectasis of the Right Lower Lobe (RLL) parenchyma with an additional pressure–displacement effect upon the Superior Vena Cava (SVC) and (R) heart sinus were detected. These findings were already indicated from the preoperatively performed chest MRI. Immediately postoperatively, the patient’s respiratory mechanics significantly improved, while the received tissue provided conditions for definitive histological classification and molecular analysis [10] (Figure 3).

**Figure 4:**
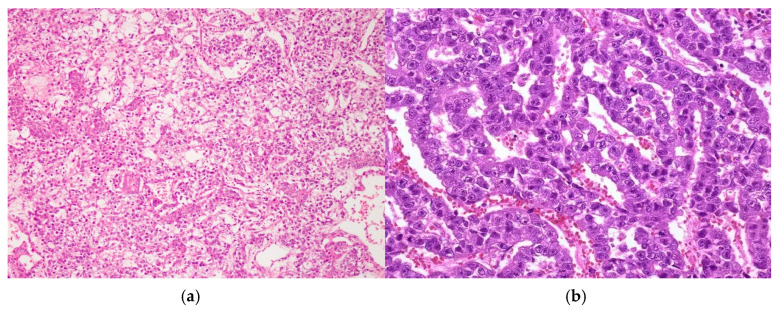
Elements of NSGCT (**a**) and embryonal mediastinal carcinoma (**b**).

**Figure 5:**
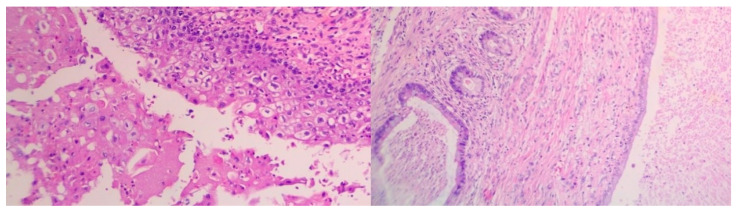
Epithelia of mediastinal teratoma.

The following histological report revealed elements of a non-seminomatous mediastinal germ-cell tumor (GCT), accompanied by findings of combined immature and immature posthuberal-type teratoma [11]. The tumor exhibited moderately cellular myofibromatoid stroma with multiple vascular structures, containing elements of immature cartilage, immature neuroepithelium, and microcystic formations, lined with columnar, cuboidal, or respiratory-type epithelium. Additionally, scattered islands of large cells with eosinophilic or basophilic cytoplasm, large nuclei, and prominent nucleoli were observed. These indications are morphologically consistent with embryonal carcinoma (Figure 4 and Figure 5).

Immunohistochemistry showed tumor cell positivity for low-molecular-weight cytokeratins and CD30, while being negative for PLAP, β-hCG, and aFP. Based on these histological and immunohistochemical findings, the patient was diagnosed with a non-seminomatous mixed germ-cell tumor (MGCT) composed of embryonal carcinoma and prepubertal teratoma (diagnosis dated 4 October 2023). Immunohistochemical staining was positive for the CD30, OCT3/4, and PLAP markers, while being negative for the CD117 marker, supporting the already-established diagnosis. Molecular analysis via next-generation sequencing detected multiple genetic alterations, notably pathogenic variants in the TP53, PTEN, and PIK3R1 markers. These findings were critical, not only in terms of successful following management but also in guiding discussions regarding potential inclusion in targeted therapy trials in the case of refractory disease.

Additional multiple bilateral testicular biopsies were negative for malignancy. Cryopreservation of sperm was undertaken prior to treatment initiation.

Given the large tumor burden and significant clinical symptomatology, chemotherapeutic treatment commenced shortly after achieving a histological diagnosis. The decision was in alignment with international guidelines (e.g., NCCN, ESMO), which recommend cisplatin-based regimens as a first-line therapy for mediastinal non-seminomatous GCTs, particularly those with embryonal carcinoma or yolk sac elements. At that time, no STM had been identified, and the assumption of chemosensitivity justified systemic treatment. The therapeutic scheme commenced with the appliance of standard 5-day first-line systemic cytotoxic chemotherapy, otherwise known as the BEP-regimen [12]. This scheme includes Bleomycin^®^ (Aeon Pharma Pvt Ltd., Mumbai, India; IFET, Rome, Italy) (30,000 IU i.v. on days 1 and 15), Cisplatin^®^ (Pfizer Hellas, Athens, Greece; Pharmanel Pharmaceuticals, Athens, Greece; IFET, Rome, Italy) (20 mg/m^2^ i.v. on days 1–5), and Etoposide^®^ (Aeon Pharma Pvt Ltd., Mumbai, India; IFET, Rome, Italy; Chemipharm Ltd., Budapest, Hungary) (100 mg/m^2^ i.v. on days 1–5) (Table 1). On the 6th day, the patient received recombinant human granulocyte colony-stimulating factor (G-CSF) to stimulate bone marrow hematopoiesis. The BEP therapeutic scheme is typically administered via 3–4 cycles, especially for non-seminomatous GCTs, offering a favorable response rate regarding gonadal tumors.

On the 21st day, the patient manifested worsening dyspnea. Laboratory examinations revealed grade 3 anemia, attributed to chemotherapy-induced myelotoxicity and leading to several red blood cell (RBC) blood transfusions. Additionally, supplemental O_2_ appliance was required via a nasal cannula at 2–3 L/min. The following cardiologic evaluation, along with a new chest CT, showed no significant acute changes compared to the most recent prior studies. Tumor markers continued to follow a declining trend. Notably, aFP levels declined, even coming close to reaching normal values, compared to the initial values, although they remained persistently elevated.

Further imaging via PET-CT revealed residual disease in the mediastinum and lymph nodes. Immunotherapy was discussed as an adjunctive strategy, but the limited PD-L1 expression and absence of microsatellite instability made the patient a suboptimal candidate [13]. The presence of residual disease in both mediastinal and osseous compartments raised concerns about incomplete response and future relapse risk. Given suboptimal results, due to the substantial loss of functional right pulmonary parenchyma, the ongoing need for supplemental oxygen, and the elevated risk of pulmonary decompensation, the continuation of Bleomycin^®^ was deemed unsafe.

Thus, the scheme was modified to VIP-Regimen, specifically, Cisplatin^®^ (20 mg/m^2^ i.v. on days 1–5) Etoposide^®^ (75 mg/m^2^ i.v. on days 1–5), and Ifosfamide^®^ (Sanofi Hellas, Athens, Greece) (1200 mg/m^2^ i.v. on days 1–5), with the additional application of Mesna^®^ (Baxter Hellas, Athens, Greece; IFET, Rome, Italy) (120 mg/m^2^ i.v. on day 1 and continuously 1200 mg/m^2^ i.v. on days 1–5) (Table 2) [14]. This is an alternative chemotherapeutic choice, used particularly in cases of poor prognosis or platinum-refractory disease, to avoid additional pulmonary toxicity. VIP-Regimen is always administered under close monitoring due to the increased eventual hematologic toxicity. During therapy, the patient developed febrile neutropenia, necessitating dose adjustments, the administration of G-CSFs, and inpatient supportive care. Further imaging controls indicated that the mass continued to significantly increase (DMax: 28 cm × 25 cm × 13 cm), with severe localized pressure effects on neighboring cardiopulmonary and vascular structures.

Clinically, we observed rapid weight loss and a progressively worsening clinical status, despite a continued decline in serum tumor markers, as well as hypoxemia (SpO_2_ 85% on FiO_2_ 21%), requiring continuous oxygen supplementation, and tachycardia.

**Figure 6:**
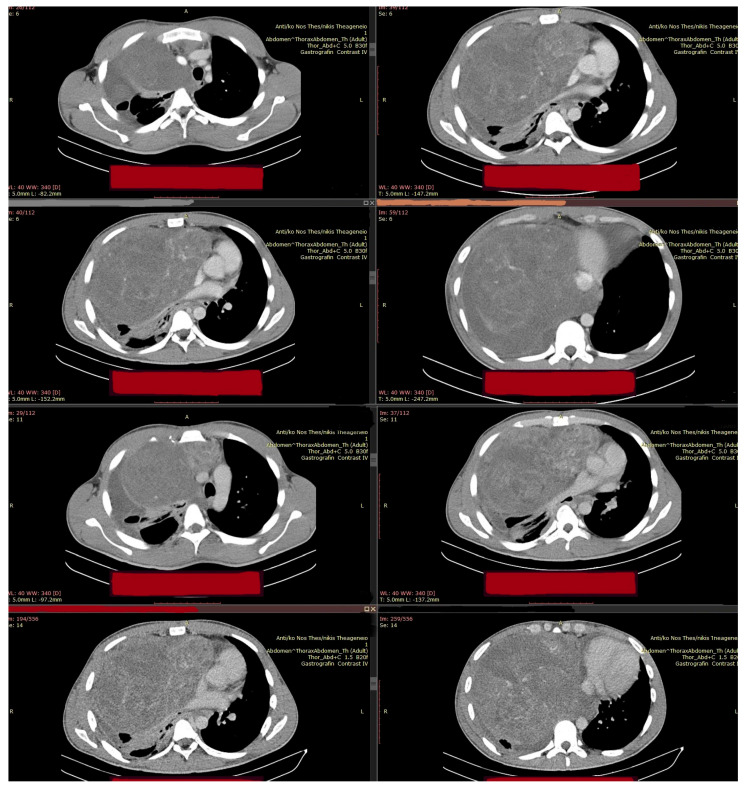
Preoperative (pro median sternotomy, extended with complementary “T-Shaped” mini anterior (R) thoracotomy) Chest-CT.

During this second line of chemotherapy, the progressive worsening of the patient’s clinical condition necessitated a third surgical intervention, focused primarily on “life-saving” requirements and current life-threatening indications [15] (Figure 6). The evolution of residual disease, following chemotherapy, demanded emergency surgical intervention, requiring radical resection, especially given the presence of teratoma and suspected chemoresistant elements on imaging. According to published reports and case series (e.g., Bokemeyer et al. [16], 2002; Kessler et al., 2004 [17]), post-chemotherapy resection is critical in achieving cure in patients with residual mediastinal masses, as viable teratoma or transformed malignancy may persist despite radiologic response. In contrast to upfront surgery, which might have been incomplete or hazardous due to the tumor bulk and vascular proximity, a successful radical resection would also provide a more definitive pathological assessment, confirming that preoperative biopsy and imaging may significantly underestimate tumor complexity.

**Figure 7:**
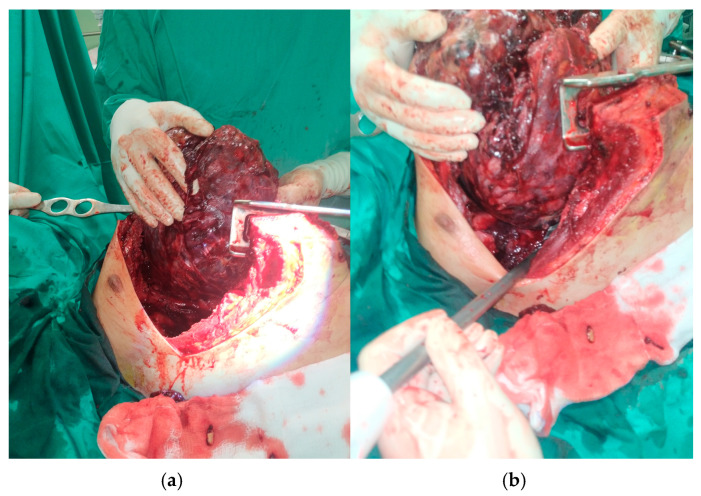
The mediastinal tumor is surgically prepared (**a**) and ready to be completely extracted via the use of a stapler (**b**).

**Figure 8:**
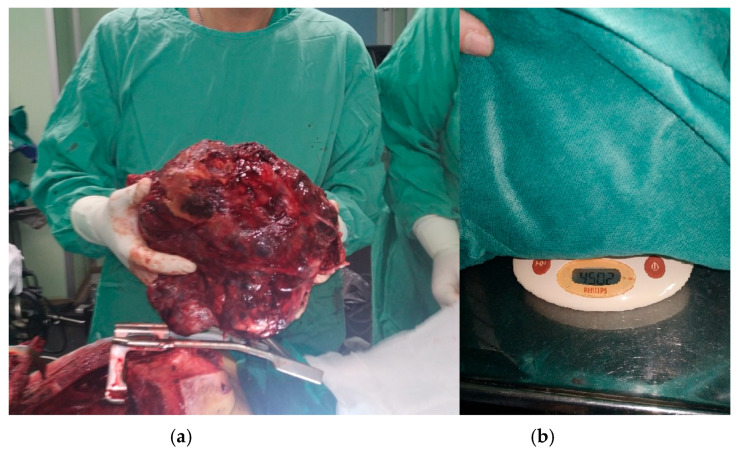
The tumor is successfully extracted (**a**) and weighed (**b**).

**Figure 9:**
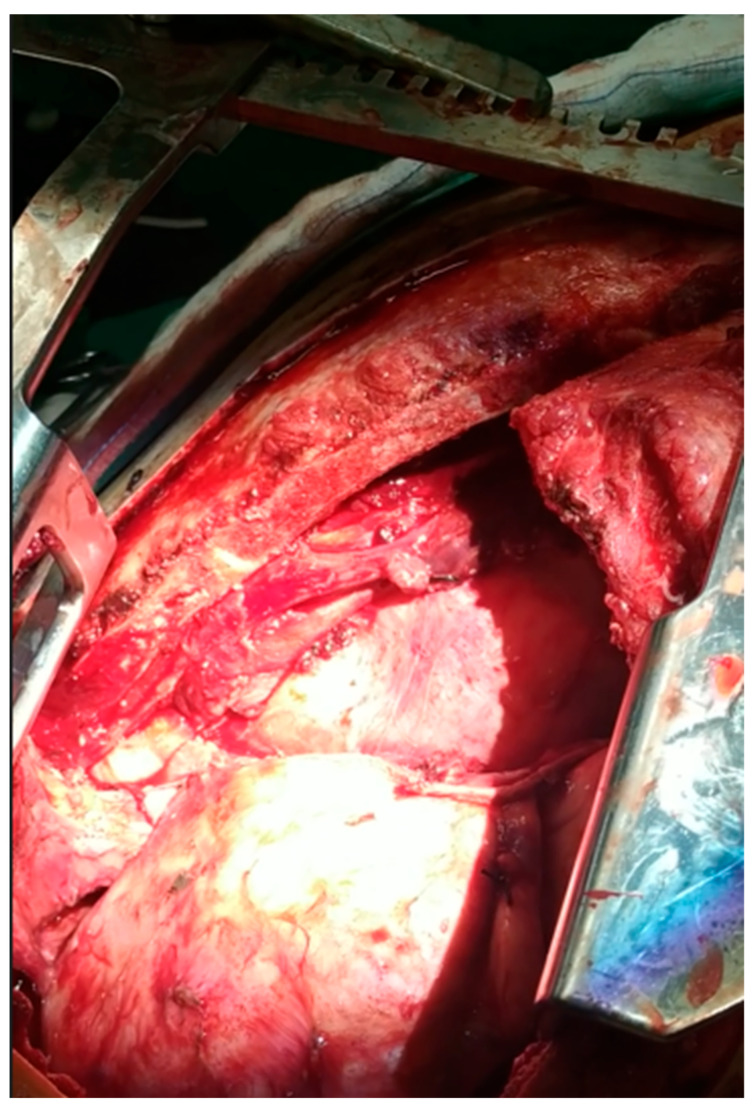
The patient’s mediastinal area after the mass’ extraction.

**Figure 10:**
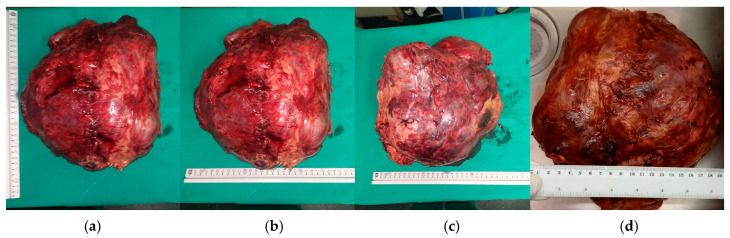
The extracted tumor is ready for histological examination. (**a**–**c**) Pre-inking grossing photograph and oblique section of the tumor (**d**).

The procedure took place via median sternotomy, extended with a complementary “T-Shaped” mini anterior (R) thoracotomy. Intraoperatively, elements of the significant pericardial adhesion of the pericardium and neighboring great vessels were detected [18] (Figure 7, Figure 8, Figure 9 and Figure 10). These findings precluded complete 100% surgical excision, achieved though significant debulking and complete extraction of the main malignant mass.

Histologically, the resected specimen exhibited

- Extensive necrosis and non-viable embryonal carcinoma, suggesting a partial chemotherapeutic response.
- Residual teratoma of postpubertal type, consistent with earlier findings.
- Scattered foci of high-grade somatic-type malignancies, including:
  ○ Rhabdomyosarcoma (Desmin+, Sarcomeric Actin+);
  ○ Chondrosarcoma (S100+);
  ○ Osteosarcoma; and
  ○ High-grade glial malignant elements, morphologically and immunohistochemically consistent with glioblastoma (GFAP+, NSE+, S100+).

This unique constellation of somatic transformation within a mediastinal GCT is exceedingly rare and poorly represented in the literature. While transformation to sarcoma (particularly rhabdomyosarcoma) has been documented, the simultaneous presence of multiple high-grade lineages, mesenchymal and glial, is almost unprecedented. Each component has distinct biological behavior and therapeutic implications:

❖ Rhabdomyosarcoma tends to be aggressive and may respond to vincristine-, actinomycin D-, and cyclophosphamide-based regimens, though its chemosensitivity is less predictable.
❖ Osteo- and chondrosarcoma components are typically chemoresistant, particularly when arising within a GCT.
❖ Glioblastoma-like elements are extremely rare in this setting and are universally aggressive, with little data supporting responsiveness to standard glioma protocols in extraneural locations.

This pathological heterogeneity explains the observed partial treatment response and underscores the challenge of managing such tumors with standard chemotherapy alone. The newly revealed results could not be discovered from the first acquired tissue samples, since the initial biopsy failed to capture the full histopathological diversity, due to anatomical limitations and the inherent sampling restriction of minimally invasive techniques. Since the following preoperative plan’s requirements changed, due to the patient’s clinical condition aggravation, the main tumor’s successful resection allowed a full and detailed further histological investigation.

**Figure 11:**
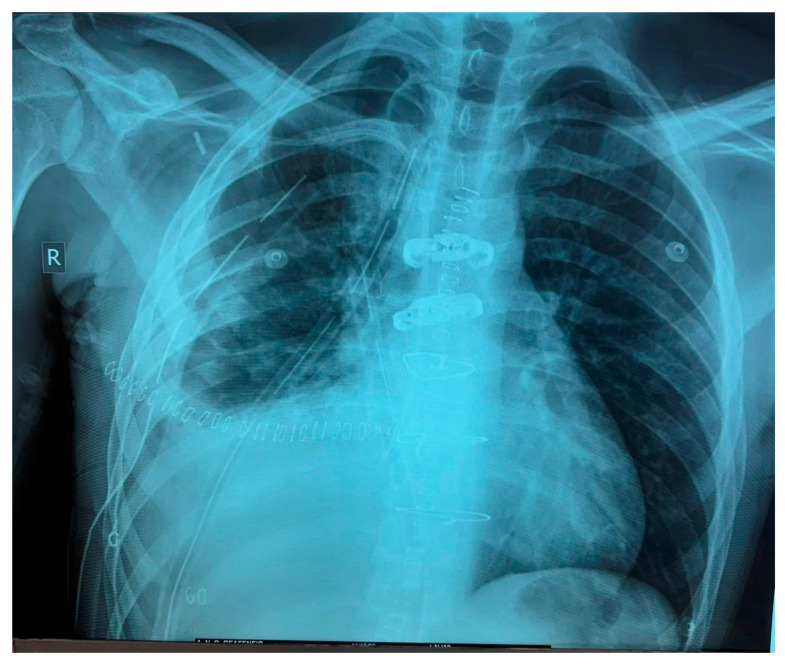
First postoperative day X-ray control (after “T-Shaped” mini thoracotomy/median sternotomy).

The patient’s postoperative status progressively improved [19]. After thorough evaluation by the multidisciplinary tumor board (MDT), the patient was placed under active surveillance (Figure 11).

The following chest CT, performed 4 months after the latter surgical procedure, demonstrated disease recurrence, characterized by a posterior mediastinal mass invading the pleura with multiple nodular lesions, exerting a pressure effect on the Inferior Vena Cava (IVC), accompanied by increasing right-sided pleural effusion, pulmonary nodules, and mediastinal lymphadenopathy [20]. Laboratory studies showed no elevation in the blood tumor markers. Given the neoplasm’s atypical and aggressive biologic behavior, along with the absence of tumor marker elevation and a strong suspicion that disease progression was driven by chemoresistant sarcomatous clones, a repeat biopsy was deemed necessary to refine the histopathologic characterization and inform treatment planning. Subsequently, the patient underwent a (R) thoracotomy, achieving multiple biopsy sampling and the partial resection of the nodular mass located in the right posterior mediastinum.

Histopathologic examination revealed findings consistent with the most recent diagnosis. Additional comprehensive molecular profiling was performed. Unfortunately, no actionable mutations were identified, while the tumor lacked the expression of biomarkers, predictive of response to targeted therapy or immunotherapy. These findings forced therapeutic management to continue with six TIP-Regimen sessions, including Cisplatin^®^ (25 mg/m^2^ i.v. on days 2–5), Ifosfamide^®^ (1500 mg/m^2^ i.v. on days 2–5, with Mesna^®^ administration at 0, 4, and 8 h at 50% of the corresponding Ifosfamide^®^ dose), and Paclitaxel^®^ (Pfizer Hellas, Athens, Greece) (250 mg/m^2^ i.v. on day 1 of each cycle), combined with Denosumab^®^ (Table 3). Prophylactic G-CSFs were administered on the 6th day of each cycle [21]. Although ENT evaluation and audiometry did not reveal any decline in auditory function, Cisplatin^®^ was omitted after the fifth cycle in order to minimize the ototoxicity risk. The patient tolerated the regimen overall well. Documented adverse events included “grade II” anorexia, “grade II” asthenia, “grade I” acral paresthesia, “grade II” diarrhea, and “grade I” tinnitus following the fifth cycle. Toxicity was graded using the Common Terminology Criteria for Adverse Events, version 5.0 (CTCAE v5.0).

**Figure 12:**
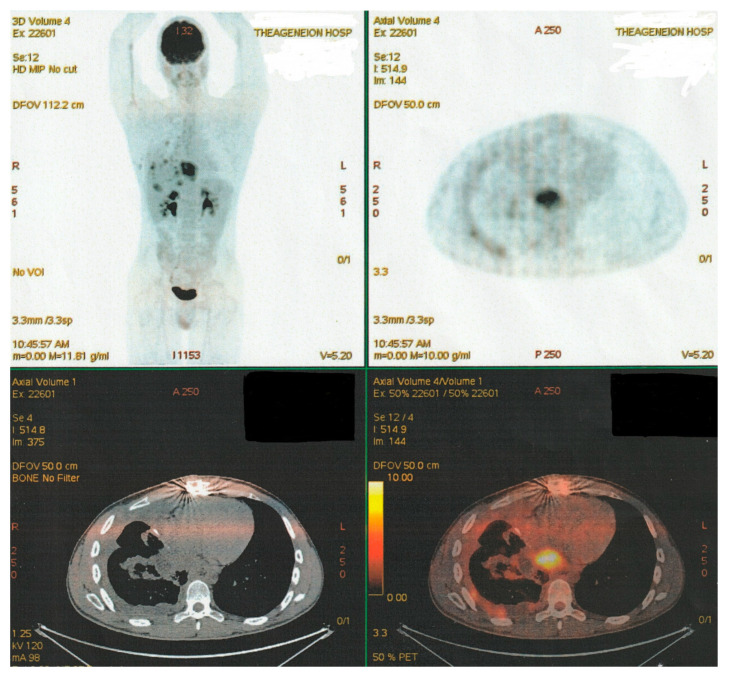
Latter PET-CT imaging control (after six “TIP-Regimen” sessions).

PET-CT imaging controls at multiple intervals, specifically after the third and sixth scheme course, revealed elements of stable disease evolution. Radiologic data indicated a decrease in metabolic activity in the mediastinum and hepatic hilar lymph nodes, hepatic parenchymal secondary lesions, the size of the retrocardiac mass and diameter of the (R) lung infiltrates, as well as the stabilization of osseous lesions {(R) femur, T_10_, L_2_, and L_3_ lesions}, though some residual metabolic activity remained persistent for a while (Figure 12).

Following additional multidisciplinary consultation at our institution, while this essay has been submitted, the patient is set to receive nine further alternating-scheme treatment courses over a time interval of four months, administered every two weeks. The VDC/IE chemotherapy regimen includes Vincristine^®^ (2 mg max i.v. on day 1), Doxorubicin^®^ (37.5 mg/m^2^ i.v. on days 1 and 2), and Cyclophosphamide^®^ (1200 mg/m^2^ i.v. on day 1), along with Ifosfamide^®^ (1800 mg/m^2^ i.v. on days 1–5) and Etoposide^®^ (100 mg/m^2^ i.v. on days 1–5). Mesna^®^ appliance will accompany the scheme (240 mg/m^2^ i.v. bolus immediately before Cyclophosphamide^®^, followed by 480 mg/m^2^ per os at 2 and 6 h post-infusion, regarding the VDC component, and 360 mg/m^2^ i.v. bolus on days 1–5, 1800 mg/m^2^ continuous infusion, in parallel with Ifosfamide^®^ and 400 mg/m^2^ i.v. bolus after completion of infusion for the IE component) (Table 4). Additionally, Dexrazoxane^®^ 750 mg/m^2^ (10× Doxorubicin^®^ dose) is set to be administered 30 min before Doxorubicin^®^ infusion, aiming to reduce the risk of anthracycline-induced cardiotoxicity. The patient will receive prophylaxis with G-CSFs after each cycle, continuing with his regular “follow-up” from his oncological team afterwards.

## 3. Discussion

Mediastinal malignant tumors are considered a rare and diagnostically challenging group of neoplasms, presenting a rather heterogeneous manifestation of histological, clinical, and overall atypical pathological features [22]. Patients are usually asymptomatic at diagnosis, especially adults, or else are suffering from mild symptomatology. Early and accurate histological identification consists of nodal examination and the consideration of important factors regarding the patient’s disease evolution and life expectancy. These tasks have a higher level of difficulty when physicians need to manage combined mediastinal masses, especially those with unexpected clinical and biological behavior [23].

Extragonadal germ-cell tumors (EGGCTs) are rare entities that arise outside of the gonads, often in midline structures such as the mediastinum, retroperitoneum, or pineal gland [24]. Primary EGGCTs are categorized into congenital, prepubertal, and postpubertal EGGCTs, with differing morphologies, types, and treatment considerations. Mediastinal EGGCTs, in particular, represent fewer than 5% of all GCTs and approximately 1–4% of all mediastinal masses. They affect primarily young male individuals between 15y. and 35y. with variable clinical symptomatology, depending on their size, location, and eventual manifestation of metastatic spread. Mediastinal EGGCTs show a strong male predominance, particularly in non-seminomatous subtypes.

Their overall incidence is estimated at 1.8 per million per year, with mediastinal localization accounting for roughly 16% of all extragonadal sites. Primary mediastinal non-seminomatous germ-cell tumors (PMNSGCTs) are associated with a significantly worse prognosis compared to their gonadal counterparts [25]. They often exhibit resistance to platinum-based chemotherapy and are frequently diagnosed at an advanced stage with bulky mediastinal disease or distant metastases, including pulmonary and skeletal involvement.

The precise pathogenesis of EGGCTs remains incompletely understood, with various theories suggesting that they arise from primordial germ cells that migrate aberrantly during embryogenesis and fail to reach the developing gonads. These cells, under certain oncogenic conditions, give rise to germ-cell tumors at extragonadal sites. The mediastinum, as a midline structure traversed by migrating germ cells, is considered a common ectopic site [26]. The overall course of mediastinal EGGCTs is often marked by fluctuations in disease burden, varying degrees of response to therapy, and the emergence of systemic complications. It is shaped not only by the underlying tumor biology but also by treatment-related toxicities and the complexities of coordinating care across multiple specialties.

The evaluative prognosis of mediastinal EGGCTs is inherently poorer compared to their gonadal counterparts, primarily due to the later stage at diagnosis, larger tumor burden, and often more aggressive histological subtypes. Several clinicopathological factors have been known to significantly influence survival outcomes and guide therapeutic decisions [27]. Key prognostic indicators include serum tumor marker levels at diagnosis (particularly aFP and β-HCG), histological subtype (pure seminomas versus non-seminomatous elements), and the presence of distant metastases, especially osseous or hepatic ones.

According to the International Germ Cell Cancer Collaborative Group (IGCCCG) classification, the presence of additional, different cytological malignant elements, such as those of embryonal carcinoma origin, place these patients within the poor-prognosis category [28]. Mediastinal embryonal carcinomas are even more rarely detected, with low prognostic indication and a relatively short accompanying life expectancy. It often represents the malignant transformation of germinal elements without gonadal focus with very few documented diagnosed reports.

The commonest histological type of mediastinal GCT is mature teratoma followed by seminoma [29]. They grow slowly as benign tumors of the anterior mediastinum, arising near the thymus gland or within the thymic parenchyma. Such cases usually involve young adults, while children are often affected, with equal allocation regarding male and female patients. They are composed of well-differentiated tissues derived from more than one of the three embryonic germ cell layers. The patients are often asymptomatic, and the tumor is discovered incidentally on chest radiographs. Large tumors may produce pathogenic symptomatology due to the compression of mediastinal structures. Rarely, these tumors may rupture or erode into adjacent structures, such as the pleural space, the pericardium, the lung, or the tracheobronchial tree.

In such incidents, pleural and/or pericardial effusions, lipoid pneumonia, or the expectoration of oily substances or hair may manifest, accompanied by spontaneous pneumothorax and acute cardiac tamponade. After typical imaging, they appear as rounded, occasionally lobulated anterior mediastinal masses, with sharply marginated borders against the adjacent lung and elements of calcification. Additional radiographic visualization of teeth is pathognomonic of teratoma, as well as the presence of fat-fluid levels, considered quite specific to the final diagnosis. Postpubertally, tissues of immature teratomas of mediastinal GCTs may transform into malignant elements, an occurrence that significantly worsens prognosis. Malignant transformation may lead to the development of hematopoietic malignancy, most commonly subtypes of acute myeloid leukemia and also carcinomas and sarcoma subtypes.

Primary sarcomas of the mediastinum are also rare and account for 2 to 8% of malignant mediastinal tumors. Patients’ clinical symptomatology is predominately respiratory, while a definite diagnosis is achieved, almost in every case, via surgical excision.

**Figure 13:**
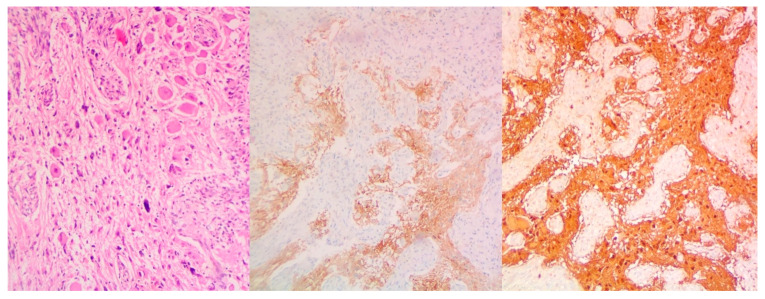
Elements of mediastinal Rhabdomyosarcoma.

**Figure 14:**
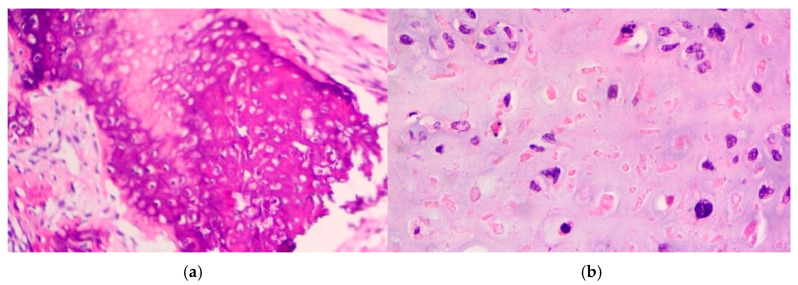
Elements of mediastinal Osteosarcoma (**a**) and Chondrosarcoma (**b**).

Mediastinal sarcomas vary histologically, depending on the primary source of malignancy. Physicians may encounter a wide specter of subtypes, such as rhabdomyosarcomas, chondrosarcomas, angiosarcomas, neurovascular sarcomas, myosarcomas, etc. Their rarity and diversity of clinical presentations increase significantly, regarding the difficulty in management, when sarcomas evolve combined with other types of mediastinal malignancies, such as EGGCTs (Figure 13 and Figure 14).

**Figure 15:**
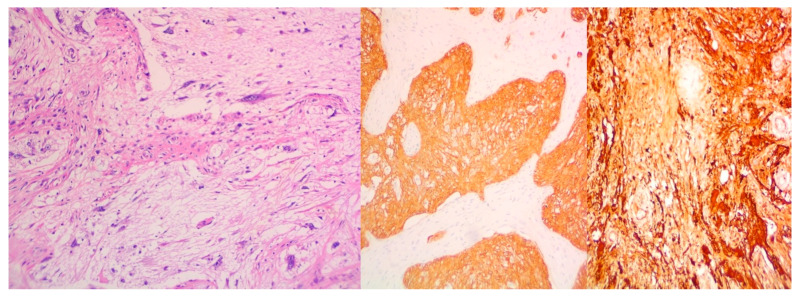
Elements of mediastinal Neuroglia and Glioblastoma.

Mediastinal gliomas and glioblastomas present another rare type of malignancy; not only does it evolve as a result of the late metastatic manifestation of primary EGGCTs but their cytological activity progresses side by side with the co-existence of teratomas, mature or immature [30]. The overall prognosis and life expectancy rates are measured inversely proportionally in comparison with the degree of tissue complexity (Figure 15).

Previous reports have described such transformations; however, this case is exceptional in demonstrating multiple, diverse elements, emphasizing that

- Somatic transformation may emerge from various germ cell lineages (teratomatous, embryonal);
- Transformation may occur along multiple differentiation pathways;
- Routine imaging or serologic monitoring alone is insufficient to capture progression in such biologically diverse tumors.

Future treatment approaches are likely to incorporate a greater degree of personalization, leveraging next-generation sequencing and molecular profiling to guide targeted therapy, although molecular instability limits current immunotherapy applicability [31]. Novel immune checkpoint inhibitors, vaccines, and adoptive T-cell therapies are being studied in refractory mediastinal malignancies with encouraging preliminary results. The role of high-dose chemotherapy followed by autologous stem cell transplantation (HDCT-ASCT) is being re-evaluated. Biomarkers still remain predictive of benefit (Table 1). There is a critical need for the development of histology-driven treatment algorithms, possibly informed by molecular profiling, as rare combined tumors may respond variably to existing chemotherapeutic or immunotherapeutic regimens.

Although current guidelines prioritize histology and serum markers in treatment planning, this case highlights the potential role of early molecular and genomic profiling (e.g., next-generation sequencing, mutational panels, DNA methylation signatures) in

✓ Detecting early signs of somatic transformation;
✓ Differentiating among components with distinct therapeutic vulnerabilities;
✓ Guiding the selection of non-standard chemotherapy protocols or other targeted agents (e.g., sarcoma- or glioma-directed therapy).

Incorporating molecular profiling at the time of initial biopsy or post-chemotherapy evaluation, even in cases with limited tissue, may allow more precise risk stratification and treatment tailoring, particularly when histological ambiguity or atypical clinical behavior is observed.

There is still an ongoing need for multicenter registries and real-world data to characterize in detail the disease’s course, particularly in rare locations such as the mediastinum, which would be invaluable in guiding management and improving long-term outcomes. Collaborative prospective studies are essential in establishing consensus guidelines, identifying novel biomarkers, and improving survival outcomes. Clinicians should maintain a high index of suspicion for combined pathology in atypical presentations, as preoperative biopsy may not capture the full tumor heterogeneity.

The evolution and comprehension of tumor biology and novel technological advances in genomics promise more precise, effective, and tolerable therapies (Table 5). Such cases reinforce the call for innovation in neoadjuvant or adjuvant strategies that can address mixed tumor biology, such as combined modality therapy tailored to the most aggressive histologic component.

Thoracic surgery interventions accompany this difficult task, whether diagnostic or therapeutic, providing the appliance of even more minimally invasive procedures, which substantially facilitate patients’ early placement on a precise management route and relief from the disease’s oncological burden. While platinum-based chemotherapy remains the standard for non-seminomatous GCTs, when the disease evolution shows a limited response to treatment, successful surgical resection is necessary to cure the patient. As demonstrated here, surgery revealed both the failure of chemotherapy to eradicate certain components and the presence of high-grade lineages that were previously undetected.

The disease’s psychosocial impact and its treatment also emerge as crucial elements of care. The patient often requires psychological support for anxiety, treatment fatigue, and social reintegration. Coordination with palliative care services may ensure early symptom management and holistic follow-up. This level of management underscores the necessity for proactive complication surveillance, interdepartmental collaboration, and tailored adjustments to maintain therapeutic momentum, without overwhelming the patient physiologically or psychologically.

The role of each institute’s MDTs remains instrumental in the task of managing evolving challenges. Weekly case conferences involving thoracic surgeons, medical oncologists, radiologists, pathologists, and critical care specialists ensure the continuous and dynamic adaptation of the treatment strategy, collaborating early in the diagnostic and therapeutic pathway to optimize outcomes. Surgical intervention, when decided on in time, favors the optimization of cytoreduction and the minimization of perioperative morbidity.

## 4. Conclusions

Mediastinal tumors represent unpredictable clinical entities due to their aggressive biological behavior, diagnostic complexity, frequent presentation with bulky, unresectable disease, and significant challenges regarding their therapeutic decision-making. The central role of multimodal treatment strategies that integrate surgical procedures, systemic chemotherapy, molecular diagnostics, and long-term multidisciplinary follow-up cannot be emphasized enough [32]. The initial approach, which involves chemotherapy followed by surgical resection, remains the gold standard, focused on stabilizing the patient clinically and relieving the mass effect, although suboptimal responses are common in numerous subtypes. Total excision, with extensive sampling, is a critical consideration in order to accurately diagnose all malignant elements and quantify the relative percentage of cell population subtypes. The incorporation of second-line regimens, along with attentive toxicity management, enables disease control, albeit with residual metabolic activity and significant treatment-related morbidity. Histopathological and genomic analyses have begun to redefine the therapeutic landscape of mediastinal malignancies, while the usage of tumor markers remains informative for risk evaluation and guiding consideration for novel therapies. While the current role of immunotherapy is limited, the number of potential applications of targeted and cell-based treatments is increasing, particularly for patients with platinum-refractory disease.

Equally important is the collaborative infrastructure provided by multidisciplinary teams. Their role in the real-time adjustment of treatment protocols, early identification of complications, and psychosocial support cannot be overstated. Each patient’s case underscores the need for personalized care plans that evolve with and reflect the realities of disease progression, therapeutic tolerance, and life impact. Future advancements will hinge on continued research into the molecular underpinnings of mediastinal tumors and the expansion of evidence-based guidelines through multicenter trials and data registries. Until then, high-quality, individualized care remains the cornerstone of effective management. This essay aims to affirm the value of precision oncology, integrative care, and early intervention in optimizing outcomes for patients in the face of constantly high diagnostic and therapeutic requirements.

This case highlights several critical considerations in the management of rare, complex mediastinal GCTs with somatic-type malignant transformation:

➢ Preoperative biopsy may underestimate tumor heterogeneity, necessitating a high index of suspicion and readiness for definitive surgery post-chemotherapy, while post-chemotherapy resection is essential in the management of mediastinal tumors, even in apparent responders. Physicians must not over-rely on limited biopsy results when imaging or clinical behavior suggest a more aggressive or atypical course.
➢ Tumor markers (aFP, β-HCG) may not reflect recurrence.
➢ Somatic transformation should be suspected in cases with atypical clinical behavior or incomplete radiologic regression. Such developments challenge existing treatment paradigms. Incomplete responses should raise suspicion and prompt the consideration of aggressive surgery.
➢ Histological surprise remains a real risk in mediastinal tumors, especially in young patients. Surgeons must be prepared for unexpected intraoperative findings and the possibility that complete resection is both diagnostic and therapeutic. Surgical resection should not be delayed if imaging suggests residual viable tumor post-chemotherapy, even when biopsy suggests a partial response. Upfront surgery or early post-chemotherapy resection may offer both therapeutic and definitive diagnostic benefits.
➢ Multimodal imaging follow-up (CT and MRI) every 3–6 months in the first two years is crucial to detect relapse early, succeeded with annual follow-up, up to 5 years, with prompt evaluation of new symptoms.
➢ Multidisciplinary collaboration is non-negotiable. Pathologists, thoracic surgeons, oncologists, and radiologists must be involved early and throughout treatment to recognize rare variants and guide dynamic management.
➢ New treatment strategies are urgently needed, potentially involving molecular characterization and targeted therapy trials for STM components.
➢ A centralized registry for rare mediastinal tumors would help consolidate clinical experience, improve diagnostic accuracy, and support future research into therapeutic innovations.

In summary, this case exemplifies the need for personalized, adaptive management strategies in rare mediastinal tumors and the essential role of complete surgical resection in optimizing outcomes where chemotherapeutic response is incomplete or unpredictable. As we have proven, rare tumor biology cannot be approached with a “one-size-fits-all” mindset, and combining aggressive local control with emerging molecular insights may offer the best outcomes for complex and heterogeneous mediastinal tumors. The importance of being open about the weaknesses in our diagnostic pathways, not only to improve future care but also to support more informed clinical decisions when textbook guidelines and real-world tumor biology do not align, is critical.

## Figures and Tables

**Table 1:** BEP-Regimen.

	Days 1 and 15	Days 1–5
Bleomycin^®^	30,000 IU i.v.	
Cisplatin^®^		20 mg/m^2^ i.v.
Etoposide^®^		100 mg/m^2^ i.v.

**Table 2:** VIP-Regimen.

	Days 1–5	Day 1	Days 1–5
Cisplatin^®^	20 mg/m^2^ i.v.		
Etoposide^®^	75 mg/m^2^ i.v.		
Ifosfamide^®^	1200 mg/m^2^ i.v.		
Mesna^®^		120 mg/m^2^ i.v.	1200 mg/m^2^ i.v.

**Table 3:** TIP-Regimen.

	Days 2–5	Day 1	Hour 0/4/8
Cisplatin^®^	25 mg/m^2^ i.v.		
Ifosfamide^®^	1500 mg/m^2^ i.v.		
Paclitaxel^®^ and Denosumab^®^		250 mg/m^2^ i.v.	
Mesna^®^			500 mg/m^2^ i.v.

**Table 4:** VDC/IE-Regimen.

	Day 1	Days 1–2	Days 1–5		
Vincristine^®^	2 mg max i.v.				
Doxorubicin^®^		37.5 mg/m^2^ i.v.			
Cyclophosphamide^®^	1200 mg/m^2^ i.v.				
Ifosfamide^®^			100 mg/m^2^ i.v.		
	Before Cyclophosphamide^®^	2 and 6 h post-infusion (VDC)	Days 1–5	Continuous infusion with Ifosfamide^®^	After (IE) infusion
Mesna^®^	240 mg/m^2^ i.v. bolus	480 mg/m^2^ i.v.	360 mg/m^2^ i.v. bolus	1800 mg/m^2^ i.v.	400 mg/m^2^ i.v. bolus
	30 min before Doxorubicin^®^ infusion				
Dexrazoxane^®^	750 mg/m^2^ i.v.				

**Table 5:** Comprehensive molecular biomarker analysis.

Biomarker	Exon/Region	Genomic Alteration	Classification	Method
HRR Status (Non-Gene Biomarker)	-	-	Not Detected	Next-Generation Sequencing (NGS)
TMB (Tumor Mutational Burden) (Non-Gene Biomarker)	-	-	7.68 muts/MB	Next-Generation Sequencing (NGS)
*PTEN* (Gene)	Exon 5	c.394G>A (p.G1325)	Likely Pathogenic	Next-Generation Sequencing (NGS)
*TP53* (Gene)	Exon 5	c.454_466del (p.P152Afs*14)	Likely Pathogenic	Next-Generation Sequencing (NGS)
*PIK3R1* (Gene)	Exon 10	c.1126G>A (p.G376R)	Likely Pathogenic	Next-Generation Sequencing (NGS)
*HIST1H2BO* (Gene)	-	c.109_110delAG	Variant of Uncertain Significance (VUS)	Next-Generation Sequencing (NGS)
*SMARCB1* (Gene)	-	c.790A>G	Variant of Uncertain Significance (VUS)	Next-Generation Sequencing (NGS)
*ROS1* (Gene)	-	c.5270G>A	Variant of Uncertain Significance (VUS)	Next-Generation Sequencing (NGS)
*KDM5A* (Gene)	-	c.2150G>T	Variant of Uncertain Significance (VUS)	Next-Generation Sequencing (NGS)
MSI (Non-Gene Biomarker)	-	Stable	MSI-Stable	Immunohistochemistry
HER2 (Non-Gene Biomarker)	-	Negative	HER2-Negative	Immunohistochemistry
PD-L1 (Non-Gene Biomarker)	-	CPS < 1%	Low Expression	Immunohistochemistry

## Data Availability

The original contributions presented in this study are included in the article. Further inquiries can be directed to the corresponding author.

## Abbreviations

aFP	Alpha-Fetoprotein
ASCT	autologous stem cell transplantation
CT	Computer Tomography
CTCAE	Common Terminology Criteria for Adverse Events
EGGCT	Extragonadal Germ-Cell tumor
FNA	Fine-Needle Aspiration
G-CSF	human Granulocyte Colony-Stimulating factor
GCT	Germ-Cell tumor
HDCT	high-dose chemotherapy
IGCCCG	International Germ Cell Cancer Collaborative Group
IVC	Inferior Vena Cava
LDH	Lactate Dehydrogenase
MDT	multidisciplinary tumor board
MGCT	Mixed Germ-Cell tumor
MRI	Magnetic Resonance Imaging
PET-CT	Positron Emission Tomography–Computed Tomography
pmNSGCT	primary mediastinal Non-Seminomatous Germ-Cell tumor
RBC	red blood cells
RLL	Right Lower Lobe
SUV	standardized Uptake Value
SVC	Superior Vena Cava
VATS	Video-Assisted Thoracic Surgery
β-HCG	beta-human Chorionic Gonadotropin
